# Does Mindfulness Mediate the Relation between Impulsiveness and Job Stressfulness Perception of Professional Drivers?

**DOI:** 10.3390/ijerph20054559

**Published:** 2023-03-04

**Authors:** Piotr Mamcarz, Paweł Droździel, Radovan Madleňák, Saugirdas Pukalskas, Sylwia Gwiazdowska-Stańczak

**Affiliations:** 1 Department of Emotion and Motivation Psychology, Katolicki Uniwersytet Lubelski Jana Pawła II, al. Racławickie 14, 20-022 Lublin, Poland; 2 Department of Sustainable Transportation and Energy Sources, Politechnika Lubelska, Nadbystrzycka 36, 20-618 Lublin, Poland; 3 Department of Communications, Žilinská Univerzita V Žiline, Univerzitná 8215/1, 010 26 Žilina, Slovakia; 4 Department of Automotive Engineering, Vilniaus Gedimino Technikos Universitetas, J. Basanavičiaus g. 28, 03224 Vilnius, Lithuania; 5 Department of Educational and Family Psychology, Katolicki Uniwersytet Lubelski Jana Pawła II, al. Racławickie 14, 20-022 Lublin, Poland

**Keywords:** mindfulness, impulsiveness, organizational factors, professional drivers, safety, perception, stress

## Abstract

(1) Background: Professional driving is a stressful occupation that requires high levels of attention and decision-making, often leading to job stress. Impulsiveness, a personality trait characterized by a tendency to act without forethought, has been associated with negative outcomes such as anxiety, stress, and risky behaviors. Mindfulness has been proposed as a potential strategy for reducing job stress in various occupational settings. However, little is known about the relationship between these variables. This study aimed to investigate the mediating role of mindfulness in the relationship between impulsiveness and job stressfulness perception among professional drivers. (2) Methods: A total of 258 professional drivers from Poland, Lithuania, and Slovakia, have completed self-report questionnaires: Impulsiveness-Venturesomeness-Empathy; Subjective Assessment of Work; Five Facet Mindfulness. (3) Results: Results indicated a positive correlation between impulsiveness and job stressfulness perception, and a negative correlation with mindfulness. Mindfulness partially mediated the relationship between impulsiveness and job stressfulness perception. Additionally, variations were identified in the perceived work environment factors and mindfulness among drivers based on their country of origin. (4) Conclusions: The findings suggest that mindfulness could be a useful approach for reducing job stressfulness perception among professional drivers with high levels of impulsiveness. Given the implications of job stressfulness for professional drivers’ health and safety, developing mindfulness interventions tailored to their specific needs could be a promising direction for future research and intervention development.

## 1. Introduction

The profession of a driver is known to be a challenging and hazardous occupation that requires specialized mental and physical fitness [1,2]. The work is burdened with a high level of responsibility not only for the driver and the vehicle but also for the safety of passengers and other road users [3]. 

Professional drivers are a group that is particularly susceptible to work stress, as their job often demands to constantly stay alert and focused [4]. Freight transport drivers typically spend long hours on the road, often driving for days at a time. They may be required to deliver goods to multiple locations, which can require navigating unfamiliar routes and dealing with traffic and weather conditions [5]. Drivers in freight transport often work alone and may be responsible for loading and unloading cargo, which can be physically demanding [6]. In contrast, professional drivers in passenger transport often have a more predictable schedule and may spend more time interacting with passengers. They may be responsible for collecting fares, providing information to passengers, and ensuring the safety and comfort of their passengers [7,8]. Drivers in passenger transport often work in urban areas, which can require navigating busy streets and dealing with traffic congestion [1]. It is worth emphasizing that a driver’s geographical location can determine their range of responsibilities, the methods for handling environmental conditions, and the prevailing driving customs in that particular area [9,10]. 

No matter the nationality or classification, drivers may face similar challenges arising due to the identical nature of psychobiological tasks associated with driving [11]. As a result of the driving process itself, stress appears, which can be reinforced by factors such as monotony, shift work, time pressure, lack of social support, and negative stimuli associated with the specific work environment [12]. Perceived work stress, or the degree to which an individual views their work as stressful, can have a significant impact on their physical and mental health, as well as their quality of life and performance [13]. Research has shown that high levels of perceived work stress can lead to burnout, decreased job satisfaction, increased absenteeism, and turnover [14,15]. It also can lead to driver fatigue, decreased attention and reaction times, as well as an increased risk of accidents [4]. In addition, professional drivers who experience high levels of perceived work stress may be more likely to engage in risky behaviors such as speeding or distracted driving [16]. 

Driving-related stress is a complex phenomenon that can be influenced by a variety of factors, including personality traits, job demands, and workplace culture [17,18]. Some personality traits are correlated with higher levels of work stress, including neuroticism, perfectionism, and Type A behavior patterns [19]. Additionally, impulsiveness, defined as a tendency to act on a whim without considering the consequences, can be associated with higher levels of work stress and risky driving behaviors [20]. Impulsiveness may make it more difficult for drivers to regulate their emotions and cope with demanding work situations [21]. On the other hand, impulsivity as a temperamental characteristic may increase the risk of PTSD [22]. The level of arousal plays an important role, as reactivity and impulsivity can modify the perception of the job environment [23]. Furthermore, impulsive behavior manifests itself not only in motor skills but also in the areas of planning, thinking, and attention functions. The greater the impulsiveness, the worse the focus on driving, and the higher the tendency to aggressive and risky behavior [24]. These relationships are found not only in young drivers but also in older ones. Regardless of age, impulsive people are more likely to act carelessly, spontaneously, and inconsistently with regulations [25,26]. Drivers with a tendency for aggressive behavior make more mistakes on the road [27]; therefore, it is timely to look for ways to improve the efficiency of work drivers, their welfare, and also to strengthen resilience resources [28]. 

One of the methods of preventing negative factors in the work environment and reduction of stress levels is mindfulness, which is a term with multiple definitions in the field of psychology [29]. Generally, mindfulness is a mental state characterized by a non-judgmental awareness of the present moment [30]. It involves being attentive to one’s thoughts, feelings, bodily sensations, and surroundings, without getting caught up in them or reacting to them. It refers to the ability to concentrate on the activity being performed in the present moment [31]. However, according to Kabat-Zinn (2003), mindfulness is “the awareness that emerges through paying attention, on purpose, in the present moment, and non-judgmentally to the unfolding of experience moment by moment” [32]. This means that mindfulness involves being focused on internal or external stimuli without getting distracted by intellectual-emotional influences. High levels of mindfulness involve objectively gathering information from the world without subjectivizing the data [33]. Mindfulness is not concerned with exploring past or future events that can affect experiences or sensations. Mindfulness can be developed and can act as a protective factor against traumatizing situations [34]. 

A popular method for reducing stress levels is mindfulness-based stress reduction (MBSR) training [35]. Research shows that mindfulness training can not only improve well-being [34,36] but also reduce aggression and anxiety levels [37,38]. Mindfulness is also associated with lower occupational stress risk and higher work engagement [39]. In addition, mindfulness is negatively correlated with the frequency of driver’s distracting activities, such as talking to passengers, texting, and technology use, and is associated with situational awareness while driving [40,41]. Studies have also found that higher levels of mindfulness are associated with fewer aggressive behaviors on the road by drivers [30]. Mindfulness can help drivers stay safe and focused on their driving, manage their fatigue, and develop better relationships with their passengers and colleagues [30,42]. Research has shown that mindfulness can help reduce impulsiveness by promoting greater self-awareness and self-regulation [43]. Furthermore, other studies provide evidence that mindfulness practice can enhance the capacity to cope with challenging working conditions [44] and change stress perception [45]. There is still little research on the impact of impulsivity on job stressfulness perception and the importance of mindfulness in relation to these variables in professional drivers [46]. This is why, based on the results presented above, the following hypotheses were formulated:

1. There is no statistically significant difference between drivers from Poland, Slovakia, Lithuania, and the perception of stressors at work.
2. There is no statistically significant difference between drivers from Poland, Slovakia, Lithuania, and mindfulness facets.
3. Mindfulness facets correlates negatively with both impulsivity and job stressfulness perception.
4. Mindfulness facets mediate the relationship between impulsivity and job stressfulness perception.

## 2. Materials and Methods

### 2.1. Measures

Three methods with diagnostic properties were used to verify the hypotheses. 

The Impulsivity Inventory (IVE) by Hans J. Eysenck and Sybil B. G. Eysenck consists of 54 items to which the subject responds by answering “yes” or “no”. The results are included on three scales: impulsivity, venturesomeness, and empathy. Impulsivity is defined here as the pathological aspect of risky behavior, deviating from the norm. It is a tendency to take risks while not anticipating the consequences of actions. The method has satisfactory psychometric properties as the reliability ranges from 0.59 for Empathy, to 0.81 for Impulsivity [47]. 

The Five Facet Mindfulness Questionnaire (FFMQ) by Ruth A. Baer et al. is used and adopted in many countries including France, Brazil, the Netherlands, Germany, Norway, China, and Chile. It consists of 39 items that measure the tendency to be mindful in daily life. The subscales of the questionnaire are observing (the ability to perceive and recognize internal or external stimuli), describing (marking internal experiences in words), acting with awareness, non-judging (referring to a non-judgmental attitude toward thoughts, sensations, or emotions), and non-reacting (referring to how distanced a person is from one’s thoughts and feelings and how quickly he or she reacts to a stimulus). It is a reliable and valid method (Cronbach’s alpha ranges from 0.75 to 0.92 for each facet) [48].

The Questionnaire for Subjective Work Evaluation by Bohdan Dudek et al. is a method that can be used to assess the subjective perception of work and to measure an individual’s sense of exposure to psychosocial occupational hazards. The tool makes it possible to assess the overall workload caused by 77 organizational and psychosocial factors, as well as to identify the group of factors that affect occupational stress. The questionnaire consists of 57 items describing various job characteristics, which cluster into the following factors: work overload, lack of rewards, insecurity caused by the organization of work, social contact, threat, physical burdens, unpleasant working conditions, lack of control, and lack of support and responsibility. Based on the total score, the level of job stressfulness perception can be determined. The value of Cronbach’s α coefficients for individual factors ranges from 0.49 to 0.83, and for the questionnaire as a whole is 0.84 [49]. 

A lie scale has also been added, which measures the need for social approval or the tendency to portray oneself favorably. It includes two yes-no selection questions taken from Eysenck Personality Inventory. Answering them inconsistently with the key undermines the subject’s sincerity [50].

### 2.2. Procedure

After obtaining approval for the study from the Ethics Committee, a test package was prepared in Polish. The questionnaires were then translated into Slovak and Lithuanian. The translations were evaluated by competent judges (people familiar with the two languages), and their suggestions were implemented. The final version was still subjected to linguistic correction. The research was conducted simultaneously in three countries: Poland, Lithuania, and Ukraine. Mixed sampling was used for the participants’ selection. Major carriers within each country were selected and requested to allow access to a chosen group of employees. In the first stage, a purposive selection of carrier companies was used; in the second stage, a random sampling method was used to target respondents. The questionnaire methods were administered directly to the drivers by the investigator, who supervised the course of the study (she informed them about the purpose of the study, assisted in obtaining consent to participate in the research, and informed them that the study could be discontinued at any time). The data collected were anonymous. As intended, 100 packets from each country were obtained; however, after removing the questionnaires with missing data, with non-diagnostic data (chose the same answer throughout the questionnaire, e.g., no or one value on a 5-point scale) or with two false answers in the lie scale, a total of 258 packets were included for further analysis. The data were statistically analyzed using IBM Statistical Package for the Social Sciences (SPSS) 25 along with the PROCESS plugin by Hayes [51].

### 2.3. Participants

The study group consisted of 258 city bus drivers from three countries: 99 from Poland, 91 from Lithuania, and 68 from Slovakia; a percentage of 10.5% were female and 89.5% were male. The average age was 38.60 years and the drivers’ mean length of service was 14.29 years. Among the respondents, the predominant educational background was secondary education (45.2%), vocational education (29%), higher education (22.6%), and primary education (3.2%). The job satisfaction of those surveyed was characterized as high (43.8%), medium (27.9%), very high (20.9%), and low (4.7%). A percentage of 40.3% of the respondents declared that they had never received a fine, 24% only had one fine, and 20.2% had three fines or more. Moreover, 79% of the drivers surveyed had never had a car accident, 30% declared that they had never had a car crash, 30% had experienced one crash, 15% had two crashes, and 10% had three crashes.

## 3. Results

To determine the data distribution, the rule of thumb was used. The skewness ranges from −0.57 to 0.28 and the kurtosis ranges from −0.62 to 0.83 so the normality of the distribution can be assumed [52]. ANOVA with Turkey HSD post hoc test was used to identify international differences. Perceptions of the work environment and its stressfulness differed statistically significantly from country to country. The individual factors that contribute to occupational stress in drivers are shown in Table 1 and Table 2.

Work overload (F = 6.47, *p* < 0.00) and Work conditions (F = 8.28, *p* < 0.00) were highest in the drivers from Slovakia and significantly differentiated them from the drivers from Poland (*p* < 0.00) and Lithuania (*p* < 0.00). In the case of Lack of rewards (F = 4.97, *p* < 0.00), Physical burdens (F = 5.43, *p* < 0.00), and Threat (F = 27.85, *p* < 0.00), there were no differences between the drivers from Poland and Slovakia, but significant differences were seen for the Lithuanian population (*p* < 0.00). Compared to the other groups, the drivers from Slovakia had the lowest sense of Control (F = 12.89, *p* < 0.00) while the drivers from Poland had the highest level of Responsibility (F = 4.99, *p* < 0.00). Lack of support (F = 0.38, *p* = 0.68), Uncertainty in work (F = 2.39, *p* = 0.09), and Social relations (F = 2.58, *p* = 0.08) are organizational stress dimensions that did not differentiate the occupational drivers from the countries studied. The highest Job stressfulness perception characterized Slovak drivers, which distinguishes them from drivers from Poland and Lithuania (F = 8.94, *p* < 0.00). 

There was no significant difference in scores on the Describing (F = 1.28, *p* = 0.28) and Non-judging (F = 1.03, *p* = 0.36) scales between drivers from different countries. The drivers from Poland had a significantly higher score on the Observing scale’ (F = 9.59, *p* < 0.00) than the drivers from Lithuania and Slovakia. Scores on the Acting with awareness scale did not differentiate between the drivers from Poland and Lithuania; however, they were significantly higher than those of Slovak drivers (F = 11.76, *p* < 0.00). For Non-reactivity, the highest level characterized the drivers from Poland, distinguishing them from the other groups (F = 8.34, *p* < 0.00). Impulsivity as a trait did not differentiate between the drivers from Poland and Lithuania (*p* = 0.14), but the professional drivers from Lithuania scored statistically significantly lower on this scale than the Slovak group (F = 3.65, *p* = 0.03). Table 3 presents the means, standard deviations, and correlations between the variables studied. 

Concerning the assumed mediation model, of all attentiveness factors, only Describing, Acting with awareness, and Non-judging correlated with Impulsiveness (the a-path) at the level of significance (*p* > 0.00); hence, these components will be included in further analyses. The c-prime path is also statistically significant (*p* > 0.00). The correlation between Job stressfulness perception and the number of accidents (r = 0.16, *p* = 0.01), bumps (r = 0.14, *p* = 0.02), and fines (r = 0.16, *p* = 0.01) also appears to be crucial. The correlations, despite being at a low level, are still statistically significant. Thus, to verify the complex mediation model, an analysis of the relationship between variables was carried out using the PROCESS Hayes plugin (version 4.1). The “4” model was tested assuming Bootstrap 5000 and confidence intervals of 95% (see Table 4).

Furthermore, the analyses showed that Acting with awareness (bootstrap mean = 0.11, 95%, CI = 0.06, 0.16) and Non-judging (bootstrap mean = 0.12, 95%, CI = 0.01, 0.08) significantly mediate the association between Impulsiveness and Job stressfulness perception. What is interesting is that the mindfulness factor “Describing” does not affect the direct relation between main variables (bootstrap mean = 0.02, 95%, CI = −0.01, 0.05). Finally, the analyses confirmed the theoretical model for the mediating role of mindfulness and the predictors of Job stressfulness perception: Impulsiveness and Acting with awareness [F(2, 255) = 10.76, *p* < 0.00, R2 = 0.08], and Impulsiveness and Non-judging [F(2, 255) = 6.86, *p* < 0.00, R2 = 0.05].

## 4. Discussion

The main aim of the presented research was to answer the question of the mediating role of mindfulness in the relationship between impulsivity and the job stressfulness perception of professional drivers [53]. In addition, goals of an exploratory nature were formulated for comparing drivers from European countries. 

The first hypothesis referring to no difference between drivers from eastern European countries and their perception of stressors at work was partially confirmed. Factor analysis with a post hoc test indicated no differences only in Responsibility, Lack of support, and Social relations, while for the other seven work-related stressors, the evaluation of their levels by drivers significantly varied. The basis for such results may be real existing differences in working conditions across countries, such as control management, rewards system, the scope of responsibilities, and law regulations [4,5]. Equally important subjective variables will be drivers’ personalities, coping strategies, and previous experiences [54]. What can affect the perception of environmental factors is how drivers understand cultural values and how they implement them [55]. Some of them will view working long hours as a sign of dedication and commitment, while others will prioritize work—life balance [56]. Perception of job stressfulness perception is also reinforced by disorders including anxiety, depression, ADHD [57,58], etc.

The second hypothesis regarding no difference between drivers from Poland, Slovakia, Lithuania, and mindfulness facets, was partially confirmed. For Describing and Non-judging, the results were consistent with the hypothesis. The mindfulness factors that differentiated professional drivers were Acting with awareness, Observing, and Non-reactivity. In all three facets, the highest scores were achieved by Polish drivers. Interestingly, the most variation was between drivers from Poland and Slovakia, even though these countries belong to the same socio-cultural bloc of Eastern Europe. One reason could be work characteristics, such as time and flexibility in schedules, and regular breaks that could allow them to engage in mindfulness practices [4]. Most likely, it is individual differences in drivers’ personalities, attitudes, and beliefs that could contribute to their mindfulness levels. For example, mindfulness can be predicted by neuroticism, conscientiousness, and openness [59], while the distribution of parameters in the population may vary depending on the country of origin [60]. Unfortunately, there is a lack of cross-cultural studies, on this topic, of professional drivers, especially in the Eastern European area.

The third hypothesis points to a negative correlation between impulsivity, job stressfulness perception, and mindfulness facets, which was confirmed partially. The correlations were weak but significant. The only moderate strength correlation is between Acting with awareness and Impulsiveness. Drivers who are highly aware of their thoughts and emotions are less likely to act impulsively because they can self-control and consider the consequences of their actions before making a decision [20,61]. Furthermore, impulsiveness can also lead to a higher level of risk-taking behavior, which can increase work stress [61,62]. By being more aware of their thoughts and feelings, individuals can make more deliberate choices about their behavior, leading to a decrease in impulsive actions [63]. Mindfulness is associated with different cognitive characteristics than impulsivity [64]; hence, raising the level of acting with awareness and non-judgmentalism significantly reduces the tendency to behave unthinkingly [65]. In addition, a deeper conscious analysis of workplace environmental factors allows for gaining more accurate data, which increases the chance of adjusting responses appropriately to the stimulus [66,67].

The last hypothesis about the mediating role of mindfulness on the relationship between impulsivity and job stressfulness perception was also confirmed partially. As it was found in the present study, only Acting with awareness and Non-judgment mediate the relationship between studied variables Job stressfulness perception and Impulsiveness. These variables correlate most strongly negatively with both Impulsiveness and Job stress perception. Describing was only correlated with Impulsiveness. Acting with awareness refers to focusing on a given activity at a given time, attentively, and watching closely for environmental stimuli on the road. Non-judgmental action is closely related to thoughts and effect felt, and as in the case of the obtained results, reduces stress levels [68]. Focusing too much on one’s internal experiences can interfere with a driver’s attentiveness on the road. He or she may be overly preoccupied with negative thoughts about work or events on the road and thus risk hazardous, reckless traffic behavior. Research into the mediating role of mindfulness in athletes has led to similar conclusions. Mindfulness significantly altered the realignment between impulsivity and anxiety [69]. The level of mindfulness is closely related positively to the quality of life, while it is negatively related to perceived stress. The greater the stress and perceived symptoms of stress, the worse the quality of life and reduced mindfulness. Importantly, mindfulness training can improve a person’s well-being and reduce perceived discomfort caused by stress [70]. Mindfulness practices can improve an individual’s overall well-being, including physical health and mental health. When an individual feels better overall, they may be better equipped to handle job stress and may be less likely to experience stress-related symptoms such as burnout [34]. Using driving simulators, it was tested what is the driving performance of people with higher levels of mindfulness perform. It turns out that mindfulness effectively blocks distractors and makes the driver much more focused on driving and, therefore, drives more safely [28]. 

Addressing such topics is intended to identify the variables that need to be improved in order to enhance drivers’ comfort and to learn about the impact of resilience factors, thus improving road safety [71]. The work of a professional driver deserves special attention, since it is highly dangerous and responsible, as confirmed in the conducted research. The driver in a given situation is simultaneously affected by many stressors, the subjective perception of which can significantly modify the strength of the impact on behavior [72]. The organizational context of work can cause undesirable consequences, leading to a decrease in safety [73].

## 5. Conclusions

In conclusion, the present study provided evidence for the mediating role of mindfulness in the relationship between impulsiveness and job stressfulness perception among professional drivers from Lithuania, Slovakia, and Poland. The findings suggest that mindfulness can be a valuable tool in reducing the negative impact of impulsiveness on job stressfulness perception among professional drivers.

However, limitations should still be considered in the present study. Firstly, the data were collected through self-report measures, which may be subject to social desirability biases. Secondly, the sample size was relatively small, and the participants were limited to professional drivers from three countries, which may limit the generalizability of the findings.

Future research could address these limitations by using alternative measures to assess mindfulness and job stressfulness perception, and by recruiting a more extensive and diverse sample of professional drivers. Moreover, future studies could investigate other potential moderators and mediators of the relationship between impulsiveness and job stressfulness perception, such as emotional regulation strategies, work demands, and social support.

Overall, this study highlights the potential benefits of mindfulness interventions in reducing the negative impact of impulsiveness on job stressfulness perception among professional drivers. Further research is needed to understand better the mechanisms underlying this relationship and to develop more effective interventions to support the well-being of professional drivers in this challenging occupation.

## Figures and Tables

**Table 1:** Differences among professional drivers from Lithuania, Slovakia, and Poland in perception of workplace environmental factors.

	Poland		Lithuania		Slovakia		F	*p*
	M	SD	M	SD	M	SD		
Work overload	19.59	5.11	19.83	5.88	22.56	6.01	6.47	0.00 **
Lack of rewards	19.30	5.99	17.98	5.45	21.01	6.71	4.97	0.01 **
Uncertainty at work	20.20	5.14	21.03	4.95	20.93	4.09	2.39	0.09
Social relations	15.03	3.02	16.06	3.69	15.60	2.96	2.58	0.07
Threat	17.51	3.77	13.92	4.05	17.67	3.32	27.85	0.00 **
Physical burdens	12.77	3.25	11.28	4.26	13.06	3.76	5.43	0.00 **
Work conditions	7.72	2.79	8.03	3.77	9.76	3.40	8.27	0.00 **
Control	16.44	2.81	16.66	3.05	14.50	2.69	12.89	0.00 **
Lack of support	7.42	2.51	7.45	2.26	7.75	2.87	0.38	0.68
Responsibility	14.89	2.82	14.08	3.03	13.47	2.92	4.99	0.01 **

Note: ** *p* < 0.01.

**Table 2:** Differences among drivers from Lithuania, Slovakia, and Poland in the mindfulness facets.

	Poland		Lithuania		Slovakia		F	*p*
	M	SD	M	SD	M	SD		
Observing	14.26	2.37	12.66	3.08	13.10	2.18	9.59	0.00 **
Describing	17.22	2.45	17.74	2.54	17.20	2.70	1.28	0.28
Acting with awareness	19.30	3.40	18.51	2.25	17.16	2.49	11.76	0.00 **
Non-judging	16.59	3.48	16.08	3.24	15.91	2.96	1.03	0.36
Non-reactivity	16.72	2.73	15.06	3.21	15.73	2.90	8.34	0.00 **

Note: ** *p* < 0.01.

**Table 3:** Means, Standard Deviations, and Intercorrelations of Study Variables.

No	Name	M	SD	1	2	3	4	5	6	7
1	Observing	13.39	0.17	1						
2	Describing	17.40	0.16	−0.18 **	1					
3	Acting with awareness	18.46	0.18	0.17 **	0.32 **	1				
4	Non-judging	16.24	0.20	−0.27 **	0.33 **	0.40 **	1			
5	Non-reactivity	15.88	0.18	0.46 **	0.25 **	0.04	−0.18 **	1		
6	Impulsiveness	5.52	0.17	−0.01	−0.24 **	−0.42 **	−0.25 **	−0.06	1	
7	Job stressfulness perception	177.39	1.59	0.03	−0.11	−0.27 **	−0.19 **	0.10	0.16 *	1

Note: * *p* < 0.05, ** *p* < 0.01.

**Table 4:** Path Analysis with Logistic Regression (PALR) and the effect of mindfulness factors (M) mediation on the relation between impulsiveness (X) and job stressfulness perception (Y) (*n* = 258).

M	X on M = a (β ^1^)	M on Y = b (β ^1^)	Direct Effect X on Y = c’ (β ^1^)	Indirect Effect of X on Y through M = a b	(SE)	Confidence Interval (95% CI)	
Acting with awareness	−0.42 **	−0.25 **	0.05	0.11	0.03	(0.06, 0.16)
Non-judging	−0.25 **	−0.16 **	0.12	0.04	0.02	(0.01, 0.08)
Describing	−0.24 **	−0.07	0.14 *	0.02	0.02	(−0.01, 0.05)

Note: * *p* < 0.05, ** *p* < 0.01. ^1^ Standardized Beta Coefficient.

## Data Availability

The data presented in this study are openly available in FigShare at https://doi.org/10.6084/m9.figshare.22211608 (accessed on 3 October 2022).
